# Dysphonia in Occupational Voice Users: Risk Factors, Causes and Socioepidemiological Profiles

**DOI:** 10.3390/medicina62020381

**Published:** 2026-02-14

**Authors:** Jasmina Stojanovic, Snezana Radovanovic, Milica Jevtic, Strahinja Krsmanovic, Marina Jovanovic, Andra Jevtovic, Snezana Babac, Mila Veselinovic, Mila Bojanovic, Sanja B. Krejovic-Trivic, Jovana Pficer Kuzmanovic, Maja Vulovic, Milos Stepovic, Nenad Relic

**Affiliations:** 1 Department of Otorhinolaryngology, Faculty of Medical Sciences, University of Kragujevac, 34000 Kragujevac, Serbia; fonijatarkg@gmail.com (J.S.); milicazjevtic@gmail.com (M.J.); skrsmanovic8@gmail.com (S.K.); marina_jovanovic@rocketmail.com (M.J.); andrajevtovic@gmail.com (A.J.); 2 Clinic for Otorhinolaryngology, University Clinical Centre Kragujevac, 34000 Kragujevac, Serbia; 3 Department of Social Medicine, Faculty of Medical Sciences, University of Kragujevac, 34000 Kragujevac, Serbia; jovanarad@yahoo.com; 4 Institute of Public Health Kragujevac, 34000 Kragujevac, Serbia; 5 ENT Clinic, Clinical and Hospital Centre Zvezdara, 11120 Belgrade, Serbia; babac.snezana@gmail.com; 6 Faculty of Special Education and Rehabilitation, University of Belgrade, 11000 Belgrade, Serbia; 7 Department of Special Education and Rehabilitation, Faculty of Medicine, University of Novi Sad, 21000 Novi Sad, Serbia; mila.veselinovic@mf.uns.ac.rs; 8 Clinic for Otorhinolaryngology and Head and Neck Surgery, University Clinical Centre of Vojvodina, 21000 Novi Sad, Serbia; 9 Medical Faculty, University of Nis, 18108 Nis, Serbia; milabojanovic@yahoo.com; 10 Clinic for Otorhinolaryngolog, University Clinical Center Nis, 18000 Nis, Serbia; 11 Department of Otorhinolaryngology, Faculty of Medicine, University of Belgrade, 11000 Belgrade, Serbia; drskt74.st@gmail.com; 12 Clinic for Otorhinolaryngology and Maxillofacial Surgery, Clinical Center of Serbia, 11000 Belgrade, Serbia; 13 Department of Medical Statistics and Informatics, School of Dental Medicine, University of Belgrade, 11118 Belgrade, Serbia; jovana57@yahoo.com; 14 Department of Anatomy, Faculty of Medical Sciences, University of Kragujevac, 34000 Kragujevac, Serbia; maja@fmn.kg.ac.rs (M.V.); stepovicmilos@yahoo.com (M.S.)

**Keywords:** dysphonia, occupational voice users, vocal fold vibration, Serbia, voice disorders

## Abstract

*Background and Objectives*: Professional voice users (PVUs) are individuals for whom the voice is the primary tool of work, and whose professional success and income largely depend on its quality. This paper’s study population predominantly consisted of occupational voice users with moderate vocal demands and the study aimed to identify risk factors and causes of voice quality and production disorders, as well as the socio-epidemiological characteristics of affected patients. *Materials and Methods*: A retrospective study was conducted in Serbia, including 145 occupational voice users aged 20–70 years who were treated for dysphonia between August 2019 and July 2024. Data collected included demographics, symptom duration, tobacco exposure, throat and nasal swab results, gastroenterological and endocrinological evaluations and information on treatment for allergic rhinitis, asthma, and dysphonia. Patients were stratified by age, profession, and cause of dysphonia into the appropriate groups. *Results*: Dysphonia is significantly more common among female occupational users. There is a significant association between the cause of dysphonia and both age and hyperthyroidism. Smoking was highly prevalent in the study population and showed associations with selected videolaryngostroboscopic parameters; however, causal inferences could not be made due to the lack of a non-smoking comparison group. No statistically significant association was observed between positive nasal or throat swabs and dysphonia, nor between allergic rhinitis or asthma and the onset of dysphonia in occupational voice users. Treatment modality varies by gender, with male occupational voice users more likely to undergo surgery and female occupational voice users more often receiving conservative therapy. Symmetric vocal fold vibrations were observed in 85.5% of participants, while regular vibrations were present in 53.1%, and insufficient glottal closure in 10.3%. Regular vocal fold vibration was significantly less frequent in patients with structural disorders and more common in individuals aged 30–39 years. Male sex showed a borderline association with reduced likelihood of symmetric vocal fold vibrations. No independent predictors of insufficient glottal closure were identified. *Conclusions*: These results support the implementation of systematic otolaryngologic examinations, combined with mandatory education on proper voice use, vocal hygiene, and the harmful effects of tobacco smoke, as measures to prevent voice disorders in occupational voice users.

## 1. Introduction

Dysphonia is a clinical symptom of impaired voice production, characterized by altered voice quality and/or increased phonatory effort, which can disrupt communication and adversely affect quality of life [1]. The term PVUs refers to individuals whose occupations require intensive verbal communication with others. This group includes, among others, singers, teachers, coaches, and clergy. Healthcare professionals represent a specific subgroup of PVUs who additionally face challenges such as communicating in noisy environments (e.g., due to medical equipment) and interacting with patients who have communication difficulties (e.g., critically ill individuals or those with hearing impairments). Occupational voice users represent a heterogeneous group with varying degrees of vocal demand, ranging from moderate occupational voice use to high-performance PVUs. Due to the high vocal demands of their work, PVUs are more likely to experience symptoms of vocal fatigue and to develop voice disorders compared to the general population [2]. It is estimated that between 50% and 80% of teachers experience dysphonia at least once in their lifetime, whereas its prevalence in the general population is 29.9%. Nearly one in two singers (46.09%) experience dysphonia at least once in their lifetime, while the prevalence among salespeople is close to 70%. This represents a significant problem, given that for PVUs, their livelihood may be compromised by dysphonia [3].

A widely accepted classification divides PVUs into four levels according to the degree of dependence of their profession on voice quality and the potential professional consequences of dysphonia: level I—PVUs for whom even minimal voice disorders may have serious professional consequences (opera singers, actors, professional singers); level II—PVUs for whom moderate voice disorders can prevent them from performing their occupational duties (teachers, politicians, clergy, broadcasters, etc.); level III—individuals who are not PVUs but for whom severe voice disorders may hinder their professional activity (lawyers, judges, physicians); level IV—individuals whose profession does not depend on voice quality and for whom voice disorders have minimal impact on work ability (office workers, laborers, salespeople) [4].

The causes of dysphonia are numerous, ranging from mild infections to life-threatening malignant diseases. Hoarseness may result from acute or chronic laryngitis, functional dysphonia, psychogenic factors, benign and malignant laryngeal tumors, vocal fold paralysis, and even malignant conditions of the hypopharynx, thyroid gland, esophagus, and bronchogenic carcinoma [5]. In general, risk factors for the development of dysphonia in PVUs can be divided into three major categories: (1) factors related to the characteristics and organization of the work process—such as long working hours, excessive use or misuse of the voice, lack of breaks, or absence of an adequate resting area; (2) factors related to the work environment—including unfavorable acoustic conditions, polluted workplace air, low humidity, and exposure to chemicals, dust, and smoke; (3) individual factors—such as age, gender, presence of allergies, hormonal influences, gastroesophageal reflux disease, laryngopharyngeal reflux, medication use, and lifestyle habits such as alcohol consumption and cigarette smoking [6,7].

Dysphonia is increasingly recognized as a complex disorder, given its multifaceted impact on the quality of life of affected individuals. It not only causes difficulties in verbal communication but has also been shown to be associated with a range of other problems, such as low self-esteem, social isolation, depression, reduced overall quality of life, and work absenteeism [8]. Work absenteeism due to dysphonia is particularly common among teachers—they are, on average, absent from work due to dysphonia five times more often than the general population. Such absences create challenges not only within the educational system but also represent a significant economic burden for the country, as frequent work interruptions sometimes require workforce replacement. It is estimated that annual costs related to absenteeism in the United States amount to 2 billion USD, whereas in Brazil the cost per person reaches 30,000 BRL annually, with the absenteeism rate—and consequently the costs—doubling every five years [6]. The estimated annual direct costs of treating patients with dysphonia are as high as 13.5 billion USD, with this figure increasing each year [9].

Several approaches are available for the treatment of patients with dysphonia, including conservative, surgical, and behavioral therapies, as well as combinations of these modalities. According to the clinical practice guidelines of the American Academy of Otolaryngology, voice therapy is recommended for the management of patients with dysphonia, as its effectiveness has been confirmed by systematic literature reviews and randomized clinical trials evaluating the benefit–risk ratio of this therapeutic modality [9].

Vocal hygiene is one of the methods used in voice therapy. It falls under indirect interventions aimed at optimizing vocal health through education and the implementation of various strategies to modify the physical environment in which the voice is used. Vocal hygiene plays a significant role in the prevention of dysphonia, rehabilitation of patients with dysphonia, and maintenance of optimal vocal health, particularly among PVUs. Traditional vocal hygiene education typically consisted of lists of “dos and don’ts,” whereas recent analyses and clinical research emphasize the importance of an individualized approach to increase therapy effectiveness. According to current perspectives, key aspects of vocal hygiene include maintaining adequate overall hydration, ensuring proper hydration of the laryngeal mucosa, recognizing symptoms of laryngopharyngeal reflux, avoiding phonotraumatic speaking patterns, and adopting appropriate vocal behaviors especially in cases of intensive voice use [10].

The aim of this study was to investigate the patterns and underlying causes of dysphonia in occupational voice users, to identify clinical and demographic factors associated with alterations in vocal fold vibratory function, and to characterize the distribution of dysphonia according to sex, age, and professional voice demands. This study further sought to explore how these factors relate to management strategies, including conservative and surgical treatment approaches.

## 2. Materials and Methods

### 2.1. Study Design, Ethical Approval, and Data Collection

This single-center, retrospective observational study was conducted at the Clinic of Otolaryngology, University Clinical Center Kragujevac, Serbia. Medical records of patients diagnosed and treated for dysphonia between August 2019 and July 2024 were reviewed using the institutional electronic database.

Eligibility criteria included occupational voice use, age between 20 and 70 years, and treatment for organic or functional dysphonia. Patients were excluded if they had malignant laryngeal tumors, phononeuroses, or were older than 70 years. After applying these criteria, a total of 145 patients were included in the final analysis.

The study protocol was approved by the Ethics Committee of the University Clinical Center Kragujevac (No. 01/18-4922), and all procedures were performed in accordance with the principles of the Declaration of Helsinki and applicable national regulations. Informed consent was obtained from all subjects involved in the study.

This study was conducted and reported in accordance with the Strengthening the Reporting of Observational Studies in Epidemiology (STROBE) guidelines for observational studies [11]. 

### 2.2. Patient Classification, Diagnostic Assessment, and Outcomes

This study included a set of sociodemographic, clinical, behavioral, and diagnostic variables collected through medical records and patient interviews.

To investigate potential age- and occupation-related differences, patients were stratified into five age groups (20–29, 30–39, 40–49, 50–59, and 60–69 years) and four PVUs categories based on vocal load demands: group 1—singers and actors; group 2—teachers, broadcasters, politicians, and clergy; group 3—lawyers, judges, physicians, and speech therapists with elevated vocal demands; group 4—salespeople and office workers.

All assessments were performed by experienced otolaryngologists following standardized clinical protocols. Each participant underwent a comprehensive, multidimensional voice evaluation, which included detailed medical and voice history, perceptual voice assessment, laryngeal examination, and videolaryngostroboscopy [12].

Vocal fold vibration symmetry refers to the degree of synchrony between the left and right vocal folds during phonation, assessed by laryngeal examination. Symmetric vibration indicates coordinated and simultaneous movement of both vocal folds, while asymmetric vibration reflects differences in timing, amplitude, or phase between the two sides. This variable was recorded as a binary parameter and categorized as 0 = no (absence of symmetry) and 1 = yes (presence of symmetry).

Vocal fold vibration regularity describes the consistency and periodicity of vocal fold oscillation during sustained phonation. Regular vibration is characterized by stable and repetitive vibratory cycles, whereas irregular vibration indicates variability in frequency or amplitude across cycles. This parameter was treated as a binary variable and categorized as 0 = irregular and 1 = regular.

Glottal closure sufficiency refers to the ability of the vocal folds to achieve adequate closure during phonation, allowing efficient sound production without excessive air leakage. Sufficient glottal closure indicates complete or functionally adequate approximation of the vocal folds, while insufficient closure reflects incomplete contact during phonation. This variable was categorized as a binary parameter, coded as 0 = insufficient and 1 = sufficient.

Diagnostic classification was based on clinical history, physical examination, and stroboscopic findings.

Causes of dysphonia were categorized into six groups: vocal fold polyps, vocal nodules, vocal fold edema, chronic laryngitis, hyperkinetic dysphonia, and other causes (hypokinetic dysphonia, vocal fold cysts, vocal fold keratosis, contact granuloma, and sulcus vocalis).

Smoking status was recorded as the number of cigarettes smoked per day and classified into six categories: non-smokers, 1–10 cigarettes/day, 11–20 cigarettes/day, 21–30 cigarettes/day, 31–40 cigarettes/day, and more than 40 cigarettes/day.

Duration of dysphonia was defined according to patient-reported symptom duration and classified into four categories: up to one month, 1–3 months, longer than three months, and intermittent hoarseness.

Throat and nasal swab results were included as a categorical variable with three categories: no pathogenic microorganisms detected, positive throat swab, and positive nasal and throat swab. Rhinitis was assessed as a binary variable and categorized as present or absent. Asthma was also recorded as a binary variable and categorized as present or absent.

Clinical diagnosis was established by an otorhinolaryngologist based on laryngoscopic findings and classified into six diagnostic categories: vocal fold polyps, vocal nodules, vocal fold edema, chronic laryngitis, hyperkinetic dysphonia, and other diagnoses.

Type of therapy was recorded as a categorical variable and classified as conservative therapy, surgical therapy, or combined therapy. Therapeutic management was individualized according to the underlying etiology, severity of vocal fold pathology, symptom duration, and occupational voice demands. Treatment strategies were categorized as conservative or surgical.

1. Conservative management was the first-line approach for patients with functional dysphonia (e.g., hyperkinetic and hypokinetic dysphonia) and benign inflammatory or phonotraumatic vocal fold lesions (e.g., vocal fold nodules, small vocal fold polyps, and sulcus vocalis). It comprised structured voice therapy conducted by a certified speech-language pathologist and/or phoniatrician, vocal hygiene education, behavioral modification, and etiology-directed medical treatment (e.g., proton pump inhibitors for reflux-related disease and antiallergic therapy when indicated). Conservative therapy was typically administered for 8–12 weeks, with monthly clinical and videolaryngostroboscopic follow-up to assess therapeutic response.
2. Surgical intervention was reserved for patients with benign structural vocal fold lesions (e.g., large vocal fold polyps, therapy-resistant nodules or cysts, vocal fold edema, and chronic laryngitis) or persistent dysphonia refractory to adequate conservative management. Indications included significant impairment of vocal fold vibratory function or glottal closure, as well as persistence of symptoms beyond three months. Surgical management consisted of microlaryngoscopic phonosurgery, performed with the aim of maximal lesion removal while preserving the layered microstructure of the vocal fold. Postoperatively, patients underwent a standardized rehabilitation protocol including short-term voice rest followed by structured voice therapy. The postoperative recovery period ranged from 4 to 8 weeks, depending on lesion characteristics and professional voice demands.

Treatment decisions were made by a multidisciplinary team consisting of experienced otolaryngologists and phoniatricians and were based on lesion type, severity of dysphonia, professional voice requirements, and response to conservative therapy, with the aim of optimizing vocal outcomes while minimizing the risk of recurrence.

Direct measures of vocal load, including daily speaking time, years of occupational voice use, microphone use, and vocal rest, were not available due to the retrospective design of the study.

### 2.3. Statistical Analysis

The dataset was reviewed for completeness prior to analysis, and no missing data were identified for the variables included in the final analyses.

All statistical analyses were performed using SPSS software (version 22.0). Categorical variables were compared using the χ^2^ test, as appropriate. A *p*-value < 0.05 was considered statistically significant, while *p* < 0.01 indicated high statistical significance. Multivariable logistic regression analysis was performed to assess factors associated with outcomes related to vocal fold vibration symmetry, vocal fold vibration regularity, and sufficient glottal closure. The outcome variables were binary: vocal fold vibration symmetry (0 = no, 1 = yes), vocal fold vibration regularity (0 = irregular, 1 = regular), and glottal closure (0 = insufficient, 1 = sufficient).

During the development of the multivariable model, a careful selection of independent variables was performed. Smoking status was not included in the logistic regression analysis due to a pronounced imbalance between smokers and non-smokers, as well as the presence of zero-frequency cells in certain categories, which resulted in model instability. The type of therapy was excluded from the analysis because it does not represent a predictor of the studied outcome but rather a clinical consequence of vocal fold vibration symmetry findings; therefore, its inclusion would not be methodologically justified.

Due to small numbers of participants and the presence of zero frequencies in specific categories, several categorical variables were regrouped into clinically and conceptually meaningful categories. Diagnoses were classified into three groups: structural changes (polyps), benign lesions (nodules and edema), and functional/inflammatory conditions (chronic laryngitis, hyperkinetic dysphonia, and other diagnoses). This classification was based on a functional and clinical rather than histopathological approach, acknowledging that certain structural changes, including polyps, may also be benign in nature.

The purpose of this categorization was to distinguish predominant morphological patterns associated with vocal function rather than to assess malignant potential. Occupations were categorized according to the degree of professional vocal load into occupational voice users (singers, actors, teachers, and broadcasters) and other voice users. This regrouping was performed to reduce the number of categories with small or zero frequencies and to ensure model stability.

Sex and age were retained in the final model as basic demographic variables of potential relevance to the studied outcome, as well as duration of dysphonia (1 = up to three months, 2 = longer than three months).

Following regrouping, cross-tabulation analyses were conducted to verify the adequacy of frequency distributions, after which binary logistic regression was applied, and results were reported as regression coefficients (B), standard errors (SE), *p*-values, odds ratios (ORs), and 95% confidence intervals (CIs). Statistical significance was set at *p* < 0.05.

Potential sources of bias inherent to the retrospective design, including selection bias and information bias related to medical record review, were acknowledged and minimized through the use of standardized diagnostic criteria and consistent data extraction procedures.

## 3. Results

Of the total 145 occupational voice users with dysphonia, 98 (67.6%) were women. The highest percentage of participants with dysphonia in our study was in the 60–69 age group (29%), followed by the 50–59 age group (25.5%), while the lowest percentage was in the youngest group, aged 20–29 years, accounting for only 11%. The largest number of patients belonged to the group of salespeople and clerks (91; 62.8%), whereas the smallest number was in the group of singers and actors (9; 6.2%). The majority reported hoarseness lasting more than three months (90; 62.1%). Nearly all participants (144; 99.3%) were smokers, with the largest subgroup (56 patients; 38.6%) smoking up to 10 cigarettes per day, and the smallest subgroup (13 patients; 9%) smoking more than 40 cigarettes per day. The most common cause of dysphonia in our study was pseudotumors—vocal fold polyps—diagnosed in 48 patients (33.1%), followed by vocal fold edema, diagnosed in 25 patients (17.2%). The least common cause of dysphonia was vocal fold nodules, identified in 15 patients (10.3%). In 139 patients (95.9%), throat and nasal swab results were negative; 3 patients (2.1%) had a positive throat swab; 3 patients (2.1%) had both positive nasal and throat swabs; and no patient had a positive nasal swab alone. Allergic rhinitis was diagnosed in 32 patients (22.1%), and allergic asthma in 13 patients (9%) (Table 1).

Table 2 presents the endovideostroboscopic findings. Endocrinological assessment was normal in 128 patients (88.3%), while 14 patients (9.7%) were diagnosed with hypothyroidism and 3 patients (2.1%) with hyperthyroidism. Gastroenterological examination revealed gastroesophageal reflux disease in 33 patients (22.8%), whereas the remaining 112 patients had normal gastroenterological findings.

A statistically significant association was identified between gender and the underlying etiology of dysphonia (*p* < 0.001). Vocal fold polyps and chronic laryngitis were more frequently diagnosed in men, whereas vocal nodules and vocal fold edema occurred predominantly in women. Age was likewise significantly associated with dysphonia etiology (*p* = 0.001). Individuals aged 20–39 years demonstrated the highest prevalence of vocal nodules, while vocal fold edema and chronic laryngitis were most commonly observed in older age groups (50–69 years). Hyperkinetic dysphonia exhibited an age-related increase, reaching its highest proportion in participants aged 60–69 years (28.6%). A significant association was also found between profession and dysphonia etiology (*p* = 0.04). Singers and actors most frequently presented with vocal fold polyps and chronic laryngitis. Elevated rates of vocal nodules and hyperkinetic dysphonia were observed among teachers, broadcasters, politicians, and clergy. Salespeople and office workers constituted the largest proportion of affected individuals overall, particularly in cases involving vocal fold polyps, edema, and chronic laryngitis (Table 3).

Of the total number of patients included in this study, 78 (53.8%) were treated conservatively, 65 (44.8%) required surgical therapy, and 2 patients (1.4%) underwent a combination of conservative and surgical treatment (Table 4). There was a statistically significant association between gender and type of treatment (*p* = 0.032). Male patients more frequently underwent surgical treatment compared with female patients (53.2% vs. 40.8%), while conservative therapy was more common among females (59.2% vs. 42.6%). Combined therapy was rare overall and observed only in male patients (4.3%).

Vocal fold vibration symmetry

Symmetric vocal fold vibrations were observed in 85.5% of participants. The multivariable logistic regression model was statistically significant (likelihood ratio χ^2^(9) = 22.69, *p* = 0.007), with moderate explanatory power (Nagelkerke R^2^ = 0.257). Goodness-of-fit was excellent (Pearson χ^2^ *p* = 0.998).

Among the predictors, male sex was associated with a lower likelihood of symmetric vibrations (OR = 0.30, *p* = 0.056), indicating a borderline significant effect. Age showed some variability, but none of the age categories were independently significant. Structural disorders (OR = 1.27, *p* = 0.720), benign reactive lesions (OR = 0.41, *p* = 0.239), occupational voice use (OR = 0.74, *p* = 0.623), and dysphonia duration ≤ 3 months (OR = 1.04, *p* = 0.939) were not significantly associated with symmetric vibrations (Table 5).

Vocal fold vibration regularity

Regular vibrations were present in 53.1% of participants. The logistic regression model was highly significant (likelihood ratio χ^2^(9) = 66.23, *p* < 0.001) with strong explanatory power (Nagelkerke R^2^ = 0.489). Pearson goodness-of-fit was adequate (*p* = 0.947).

Significant predictors included structural disorders, which were strongly associated with lower likelihood of regular vibrations (OR = 0.03, *p* < 0.001), and age 30–39 years showed higher odds of regular vibrations compared with the reference group (OR = 5.33, *p* = 0.023). Other factors, including male sex (OR = 0.55, *p* = 0.257), age 20–29 (OR = 2.58, *p* = 0.237), age 40–49 (OR = 1.55, *p* = 0.534), age 50–59 (OR = 1.48, *p* = 0.490), occupational voice use (OR = 0.68, *p* = 0.461), benign reactive lesions (OR = 0.80, *p* = 0.698), and dysphonia duration ≤ 3 months (OR = 1.57, *p* = 0.352) were not significant (Table 6).

Glottal closure

The overall model had borderline statistical significance (likelihood ratio χ^2^(9) = 18.29, *p* = 0.051). The Nagelkerke R^2^ was 0.219, indicating limited explanatory power. Goodness-of-fit testing showed adequate model calibration (deviance *p* = 0.909).

Although diagnostic group significantly contributed to the model as a whole, none of the individual predictors demonstrated stable independent associations with insufficient glottic closure. The low prevalence of insufficient closure likely reduced statistical power.

Insufficient glottic closure was rare, observed in only 10.3% of participants. The logistic regression model had borderline statistical significance overall (likelihood ratio χ^2^(9) = 18.29, *p* = 0.051) with limited explanatory power (Nagelkerke R^2^ = 0.219). Deviance goodness-of-fit indicated adequate calibration (*p* = 0.909).

No predictors were independently significant. Benign reactive lesions showed higher odds of insufficient glottic closure (OR = 5.02, *p* = 0.070). Age 50–59 (OR = 3.10, *p* = 0.136) and male sex (OR = 1.51, *p* = 0.647) suggested a possible positive trend, but were not statistically significant (Table 7).

## 4. Discussion

This study included 145 individuals with dysphonia. More than two-thirds of all participants (98 participants; 67.6%) were women. The largest number of PVUs with dysphonia were aged 60 to 69 years (42; 29%), followed by the 50–59 age group (37; 25.5%), while the fewest participants were in the 20–29 age group (16; 11%). Statistical analysis revealed a significant difference in the occurrence of dysphonia by gender, with dysphonia being significantly more common in women (*p* = 0.027), whereas no statistically significant difference was found regarding age. Literature data also show that dysphonia is considerably more frequent in female PVUs. Previous studies have demonstrated that female teachers are more prone to dysphonia compared to their male counterparts. This gender difference in the prevalence of dysphonia has also been observed in other occupational voice users, such as singers and call center workers [13]. Authors from Brazil conducted a systematic review analyzing the prevalence of occupational voice disorders among PVUs. Based on 73 studies, they found a total of 63,126 PVUs with dysphonia, of whom 42,422 were women (67.2%), a percentage nearly identical to the female prevalence in our study [14]. Several reasons are believed to underlie the higher incidence of dysphonia in female PVUs. It is traditionally assumed that women speak more, although there is no scientific evidence supporting this claim. Additionally, some professions are predominantly occupied by women—for example, the majority of teachers, salespeople, and call center workers are female [3,12]. Table 1 shows the professions of participants included in our study—the majority were salespeople and clerks (62.8%), while singers and actors comprised the smallest group (6.2%). No statistically significant association was found between profession and the occurrence of dysphonia (*p* = 0.56).

Cigarette smoking not only has a harmful effect on the voice and voice-related quality of life [15], but there is also a significant association between smoking and the development of both benign vocal fold lesions and laryngeal precancerous conditions [16]. Smoking was highly prevalent in our study population and showed associations with selected videolaryngostroboscopic parameters; however, causal inferences could not be made due to the lack of a non-smoking comparison group. Of the total number of patients with chronic laryngitis, 90.5% reported smoking more than 20 cigarettes per day, while the remaining 9.5% smoked between 11 and 20 cigarettes per day. A similar situation was observed among patients diagnosed with vocal fold edema—88% of them smoked more than 20 cigarettes per day, while the remaining 12% smoked between 11 and 20 cigarettes per day. In other words, in these two groups, there were no patients who were non-smokers or who smoked fewer than 10 cigarettes per day, which potentially implies that higher smoking intensity is a significant risk factor for the development of vocal fold edema and chronic laryngitis.

Statistical analysis of the causes of dysphonia by gender in our study showed that there is a highly significant difference (*p* = 0.000), namely that polyps and chronic laryngitis occur significantly more often in men, whereas nodules and edema are significantly more common in women. These findings are consistent with the results of a retrospective analysis conducted by Brunner et al. in Austria, in which they examined the causes of hoarseness in 535 patients. They found that cysts, nodules, and vocal fold edema occurred significantly more often in women, while men more frequently had polyps and granulomas [17]. Similar results were reported by authors from India, Nerurkar et al., who stated that cysts and vocal fold nodules are more often diagnosed in women, whereas vocal fold polyps, as well as paresis/paralysis of the vocal folds resulting from premalignant and malignant laryngeal changes, are more common in men. The more frequent occurrence of vocal fold nodules in women is explained by the fact that they usually develop as a result of constant excessive use and misuse of the voice, leading to increased friction between the vocal folds. In women, the higher frequency of vocal fold vibrations results in greater mechanical load on the free edge of the vocal folds, which activates subepithelial fibroblasts and causes excessive deposition of collagen fibers along the free edge. In contrast, the main risk factor for the formation of vocal fold polyps is smoking, often combined with a single episode of intense vocal strain. Phonation at lower pitches increases stress in the deeper layers of the lamina propria, which can cause rupture of blood vessels, leading to hemorrhage. All of these factors contribute to the higher incidence of vocal fold polyps in men [18]. The results of our study also show that there is a highly significant difference in the occurrence of dysphonia with respect to age (*p* = 0.001), as well as a statistically significant difference (*p* = 0.04) in the causes of dysphonia in relation to profession (Table 6). In our study, nodules were diagnosed mainly in younger and middle age groups—almost two-thirds of the respondents diagnosed with nodules were between 20 and 39 years of age; vocal fold polyps were diagnosed across all age groups, predominantly in middle-aged individuals; while edema and chronic laryngitis were most often diagnosed in older age groups. A study conducted in the USA confirms that vocal fold nodules occur significantly more often at a younger age compared to polyps, among singers of both sexes [19]. Brunner et al. also reported in their study that there is a statistically significant difference in the causes of dysphonia in relation to age. Their results show that vocal fold nodules are most often diagnosed in children and adolescents, polyps in about half of cases are diagnosed in middle age, and vocal fold edema is diagnosed in more than two-thirds of cases (almost 70%) in older age groups [17]. Vocal load is a well-established etiological factor in dysphonia; however, direct quantitative measures of vocal overuse were not available in this study. Occupational category was therefore used as a surrogate indicator of potential voice exposure, which may not fully capture individual variability in vocal load.

The findings of a recent study indicate that vocal fatigue, considered an early indicator of more severe voice disorders and dysphonia, is significantly more prevalent among teachers with gastroesophageal reflux disease and allergies, whereas common colds, laryngitis, and sinusitis are not associated with an increased incidence of dysphonia or vocal fatigue [20]. Another investigation reported that singers with allergic rhinitis experience substantial limitations in their professional activities, highlighting the importance of adequate treatment to prevent vocal fatigue and the onset of hoarseness [21]. Our findings are partially concordant with these observations. In the present study, neither allergic rhinitis nor allergic asthma was significantly associated with dysphonia in occupational voice users, and no association was observed between a positive nasal and/or throat swab and the presence of dysphonia. Notably, gastroesophageal reflux disease was not directly associated with dysphonia when considered as a clinical symptom; however, it emerged as a highly significant risk factor for chronic laryngitis (*p* = 0.001). This distinction underscores the importance of differentiating dysphonia as a functional voice symptom from chronic laryngitis as a defined laryngeal diagnosis, through which reflux-related pathology may indirectly contribute to voice impairment via inflammatory and structural alterations of the laryngeal tissues [22,23]. Regarding thyroid disorders, previous studies have reported various voice alterations, although the affected vocal parameters differ considerably and remain inconsistent across investigations [24]. In line with these observations, our results demonstrated a statistically significant association between hyperthyroidism and the etiology of dysphonia (*p* = 0.018), with hyperkinetic dysphonia occurring more frequently in patients with hyperthyroidism than in healthy individuals or those with hypothyroidism.

Analysis of the applied therapeutic modality in relation to gender revealed that surgical treatment was administered in more than half of the cases (53.2%) among men, while women were treated conservatively in nearly 60% of cases. Statistical analysis showed a significant difference in treatment approach based on gender (*p* = 0.032), indicating that male individuals with dysphonia are more likely to undergo surgical treatment, whereas female are more likely to receive conservative therapy. The present study used multivariable logistic regression to identify independent predictors of vocal fold vibration symmetry, vibration regularity, and glottal closure, providing insights into factors influencing distinct vibratory characteristics of the vocal folds. Male sex showed a borderline association with reduced likelihood of symmetric vocal fold vibrations, suggesting potential anatomical or phonatory differences that may influence treatment planning. Vocal fold vibration regularity was strongly affected by structural disorders, highlighting the importance of lesion type in guiding management, while patients aged 30–39 years tended to have more regular vibrations, possibly reflecting greater vocal resilience. Insufficient glottal closure was rare, and no independent predictors reached significance, indicating that multiple factors, including subtle neuromuscular control, may affect closure. These findings emphasize that both structural pathology and patient characteristics should be considered when selecting conservative versus surgical interventions for dysphonia.

Several studies indicate that chronic exposure to tobacco smoke can alter the biomechanical properties of the vocal folds, leading to mucosal changes and impaired vibratory symmetry [25,26]. Diagnosis emerged as an independent predictor of vibration regularity, precisely the structural disorders and age group of 30–39 years. This finding is consistent with the pathophysiology of benign phonotraumatic lesions, which are known to disrupt the layered microstructure of the vocal fold and interfere with periodic vibratory patterns [27,28]. Structural lesions directly disrupt normal mucosal wave propagation, while individuals aged 30–39 years may experience altered vibration regularity due to peak vocal load demands despite generally preserved vocal fold biomechanics, explaining why diagnosis and this age group independently predict vibration regularity.

No significant independent predictor of insufficient glottal closure was found. Insufficient glottal closure likely reflects a complex interplay of anatomical features, vocal fold lesions, subtle neuromuscular control, and compensatory phonatory mechanisms, which may explain why none of the individual variables—such as sex, age, diagnosis, professional voice use, or dysphonia duration—demonstrated a significant independent effect. The low prevalence of inadequate glottal closure in the sample further limited statistical power, making it difficult to detect potential associations

Although the study population met formal criteria for PVUs, most participants belonged to PVUs Level IV, characterized by moderate occupational voice demands. Therefore, the results primarily reflect dysphonia patterns in this subgroup and should not be generalized to elite PVUs with high vocal load.

This study has several limitations that should be considered when interpreting the findings. First, the retrospective single-center design may introduce selection and information bias and limit the generalizability of the results beyond comparable clinical settings. Second, the extremely high prevalence of smokers in our cohort may partly reflect elevated smoking rates in the underlying population as well as the clinical profile of patients presenting with voice complaints. Nevertheless, this imbalance substantially reduced internal comparability and precluded a reliable evaluation of smoking as an independent risk factor; therefore, the observed associations should be interpreted with caution. Third, the absence of direct measures of vocal load such as daily speaking duration, cumulative occupational voice use, microphone utilization, or structured vocal rest introduces the possibility of residual confounding. The lack of standardized patient-reported outcome measures, such as the Voice Handicap Index, further limits the assessment of the functional and quality-of-life impact of dysphonia. Finally, given the single-center tertiary-care setting, treatment decisions, particularly regarding surgical intervention, may reflect center-specific clinical practices, which could introduce a degree of treatment-selection bias. Nevertheless, this study provides valuable real-world clinical data from a relatively large cohort of occupational voice users and offers a comprehensive multidimensional assessment of dysphonia, contributing to a better understanding of its clinical and etiological characteristics in routine practice.

## 5. Conclusions

This study provides insight into the patterns of vocal fold vibratory function in occupational voice users based on our cohort of 145 patients. In our sample, symmetric vocal fold vibrations were frequent, with male sex showing a borderline tendency toward reduced symmetry. Vibration regularity was notably influenced by structural lesions and was higher in participants aged 30–39 years, highlighting the role of lesion type and age-related functional changes in this group. Insufficient glottal closure was uncommon, and no individual factors reached statistical significance, suggesting that multiple subtle neuromuscular and anatomical mechanisms may underlie this finding. The distribution of dysphonia causes in our cohort reflected sex, age, and profession specific patterns: polyps and chronic laryngitis predominated in men, while nodules and edema were more common in women; younger participants had more nodules, and older participants more edema and chronic laryngitis. Consistent with these patterns, surgical treatment was more frequent among men, reflecting structural pathology, whereas conservative management was more common in women. These findings from our cohort underscore the importance of individualized assessment and management strategies and support targeted preventive measures, such as routine voice screenings and education on vocal hygiene, in vocally demanding professions. Future prospective studies incorporating direct measures of vocal load are required to better elucidate the relationship between occupational voice use and dysphonia.

## Figures and Tables

**Table 1:** Summary of Participant Characteristics (Profession, Hoarseness Duration, Dysphonia Causes, and Allergic Conditions).

Characteristic	Category	N	%
Profession	Singers and actors	9	6.2
	Teachers, broadcasters, politicians, clergy	29	20.0
	Lawyers, judges, physicians, speech therapists	16	11.0
	Salespeople and office workers	91	62.8
Duration of hoarseness	Up to one month	17	11.7
	1–3 months	12	8.3
	Longer than 3 months	90	62.1
	Intermittent	26	17.9
Cause of dysphonia	Vocal fold polyps	48	33.1
	Vocal nodules	15	10.3
	Vocal fold edema	25	17.2
	Chronic laryngitis	21	14.5
	Hyperkinetic dysphonia	21	14.5
	Other causes *	15	10.3
Allergic conditions	Allergic rhinitis—Yes	32	22.1
	Allergic rhinitis—No	113	77.9
	Allergic asthma—Yes	13	9.0
	Allergic asthma—No	132	91.0
Total		145	100

* hypokinetic dysphonia, vocal fold cysts, vocal fold keratosis, contact granuloma, and sulcus vocalis.

**Table 2:** Endovideostroboscopic findings.

	Mucosal Wave (N)	%		Symmetry of Vocal Fold Vibration (N)	%	Regularity of Vocal Fold Vibration (N)	%	Sufficient Glottic Closure (N)	%
Present	59	40.7							
Reduced	64	44.1	Yes	124	85.5	77	53.1	15	10.3
Increased	22	15.2	No	21	14.5	68	46.9	130	89.7
Total	145	100.0	Total	145	100.0	145	100.0	145	100.0

**Table 3:** Cause of dysphonia in relation to gender, age, and profession of participants.

			Diagnosis						*p*-Value
			Vocal Fold Polyps	Vocal Nodules	Vocal Fold Edema	Chronic Laryngitis	Hyperkinetic Dysphonia	Other	
Gender	Male	N	20	0	1	16	4	6	0.000 *
		%	42.6	0.0	2.1	34.0	8.5	12.8	
	Female	N	28	15	24	5	17	9	
		%	28.6	15.3	24.5	5.1	17.3	9.2	
Age (years)	20–29	N	6	4	0	0	4	2	0.001 *
		%	12.5	26.7	0.0	0.0	19.0	13.3	
	30–39	N	8	5	0	3	5	5	
		%	16.7	33.3	0.0	14.3	23.8	33.3	
	40–49	N	13	3	1	3	4	0	
		%	27.1	20.0	4.0	14.3	19.0	0.0	
	50–59	N	12	2	13	6	2	2	
		%	25.0	13.3	52.0	28.6	9.5	13.3	
	60–69	N	9	1	11	9	6	6	
		%	18.8	6.7	44.0	42.9	28.6	40.0	
Profession	Singers and actors		3	1	1	2	1	1	0.04 *
	Teachers, broadcasters, politicians, and clergy		11	6	2	2	4	4	
	Lawyers, judges, physicians, and speech therapists		4	5	0	2	4	1	
	Salespeople and office workers		30	3	22	15	12	9	

* *p*-value less than 0.05, indicates statistical significance.

**Table 4:** Treatment modalities according to gender.

			Type of Treatment			Total	*p*-Value
			Conservative	Surgical	Combined		
Gender	Male	N	20	25	2	47	0.032 *
		%	42.6	53.2	4.3	100.0	
	Female	N	58	40	0	98	
		%	59.2	40.8	0.0	100.0	
Total		N	78	65	2	145	
		%	53.8	44.8	1.4	100.0	

* *p*-value less than 0.05, indicates statistical significance.

**Table 5:** Predictors of vocal fold vibration symmetry identified by multivariable logistic regression analysis.

Predictor	B	SE	*p*-Value	Odds Ratio	95% CI for OR
Male sex	−1.202	0.629	0.056	0.30	0.09–1.03
Age 40–49	−0.580	0.657	0.377	0.56	0.15–2.03
Age 50–59	0.593	0.640	0.354	1.81	0.52–6.34
Occupational voice user	−0.307	0.624	0.623	0.74	0.22–2.50
Structural disorders	0.239	0.667	0.720	1.27	0.34–4.69
Benign reactive lesions	−0.882	0.749	0.239	0.41	0.10–1.80
Dysphonia ≤ 3 months	0.043	0.564	0.939	1.04	0.35–3.16

B = regression coefficient; SE = standard error; OR = odds ratio; 95% CI = 95% confidence interval; *p* < 0.05, statistically significant; reference category: no.

**Table 6:** Predictors of vocal fold vibration regularity identified by multivariable logistic regression analysis.

Predictor	B	SE	*p*-Value	Odds Ratio	95% CI for OR
Male sex	−0.605	0.533	0.257	0.55	0.19–1.55
Age 20–29	0.948	0.801	0.237	2.58	0.54–12.41
Age 30–39	1.673	0.735	0.023 *	5.33	1.26–22.48
Age 40–49	0.437	0.702	0.534	1.55	0.39–6.12
Age 50–59	0.391	0.566	0.490	1.48	0.49–4.48
Occupational voice user	−0.383	0.519	0.461	0.68	0.25–1.89
Structural disorders	−3.480	0.624	<0.001 *	0.03	0.01–0.11
Benign reactive lesions	−0.226	0.582	0.698	0.80	0.26–2.50
Dysphonia ≤ 3 months	0.449	0.482	0.352	1.57	0.61–4.03

B = regression coefficient; SE = standard error; OR = odds ratio; 95% CI = 95% confidence interval; * *p* < 0.05, statistically significant; reference category: no.

**Table 7:** Predictors of glottal closure identified using multivariable logistic regression analysis.

Predictor	B	SE	*p*-Value	Odds Ratio	95% CI for OR
Male sex	0.408	0.891	0.647	1.51	0.26–8.63
Age 50–59	1.130	0.758	0.136	3.10	0.70–13.69
Occupational voice user	−0.198	0.757	0.793	0.82	0.19–3.61
Structural disorders	−0.428	0.967	0.658	0.65	0.10–4.33
Benign reactive lesions	1.614	0.891	0.070	5.02	0.88–28.79
Dysphonia ≤ 3 months	−0.584	0.739	0.429	0.56	0.13–2.37

B = regression coefficient; SE = standard error; OR = odds ratio; 95% CI = 95% confidence interval; *p* < 0.05, statistically significant.

## Data Availability

The original contributions presented in this study are included in the article. Further inquiries can be directed to the corresponding author.
